# A vessel-guided multi-task deep learning framework with visual interpretability for simultaneous retinal vessel segmentation and multi-disease classification from fundus images

**DOI:** 10.3389/fmed.2026.1799745

**Published:** 2026-04-17

**Authors:** Qi Li, Liming Tao

**Affiliations:** 1 Department of Ophthalmology, The Second Affiliated Hospital of Anhui Medical University, Hefei, China; 2 Department of Refractive Surgery, Nanhai Aier Eye Hospital, Foshan, China

**Keywords:** deep learning, explainable AI, fundus photography, multi-task learning, ocular disease diagnosis, vessel segmentation

## Abstract

**Introduction:**

Retinal diseases represent a leading cause of visual impairment and blindness worldwide, with early and accurate diagnosis being crucial for preventing irreversible vision loss. Although deep learning techniques have achieved significant advances in fundus image analysis, existing methods predominantly focus on single tasks, treating vessel segmentation and disease diagnosis as independent problems without fully leveraging their intrinsic relationships. Furthermore, the lack of transparency in deep model predictions limits clinical adoption; while full interpretability remains an open challenge, post-hoc techniques such as Grad-CAM can provide partial transparency by highlighting influential image regions.

**Methods:**

This study presents V-MNet, a vessel-guided multi-task deep learning framework that simultaneously achieves retinal vessel segmentation and multi-disease classification, and provides visual transparency through Grad-CAM-based class activation mapping to support clinical decision-making. The framework comprises three core modules: a shared encoder extracts multi-scale feature representations; a segmentation decoder employs a U-Net-style architecture to generate vessel masks; and a classification decoder incorporates an innovative vessel-guided mechanism that explicitly transfers structural priors from the segmentation branch to the classification task, enabling the model to precisely localize pathological regions. Concurrently, an integrated Grad-CAM module generates post-hoc class activation maps for each disease category, highlighting spatially relevant lesion regions for clinician review. Comprehensive experiments were conducted on four public datasets-RFMiD, ODIR-5K, DRIVE, and EyePACS-light-v2.

**Results:**

Experiments demonstrate that V-MNet achieves a Dice coefficient of 0.831 and AUC of 0.985 for vessel segmentation tasks, and an average AUC of 0.978 with F1-score of 0.935 for multi-disease classification tasks, significantly outperforming single-task baseline models and existing state-of-the-art methods. Ablation studies systematically quantify the performance contributions of multi-task learning and the vessel-guided mechanism, confirming the effectiveness of the framework's core innovations.

**Discussion:**

V-MNet demonstrates broad application potential as a computer-aided diagnostic tool by jointly leveraging vascular structure and disease pathology for superior performance and visual transparency. The vessel-guided multi-task design effectively exploits the intrinsic relationship between vessel segmentation and disease classification, while the integrated Grad-CAM module addresses the lack of model transparency, facilitating clinical adoption and supporting clinical decision-making.

## 1 Introduction

Retinal diseases constitute one of the leading causes of visual impairment and blindness globally. According to the World Health Organization (WHO), at least 2.2 billion people worldwide have near or distance vision impairment, and at least 1 billion of these cases could have been prevented or have yet to be addressed. Among those with distance vision impairment or blindness, approximately 94 million are affected by cataract, 7.7 million by glaucoma, 3.9 million by diabetic retinopathy (DR), and 8 million by age-related macular degeneration (AMD) (1). Taking diabetic retinopathy as an example, among the approximately 537 million adult diabetic patients globally, over one-third will develop varying degrees of DR (2). As the primary cause of irreversible blindness, glaucoma is projected to affect 112 million patients worldwide by 2040 (3). Without timely diagnosis and treatment, these diseases may lead to irreversible vision loss or even complete blindness, not only severely compromising patients' quality of life but also imposing substantial economic burdens on families and society. Therefore, early screening and accurate diagnosis of fundal diseases hold paramount clinical significance for preventing visual impairment and improving patient outcomes.

Fundus photography, as a non-invasive, relatively low-cost, and readily accessible imaging modality, has become an essential tool in ophthalmic clinical diagnosis (4). Compared to high-cost examination methods such as optical coherence tomography (OCT) and fluorescein fundus angiography (FFA), fundus photography equipment is portable and simple to operate, making it particularly suitable for large-scale population screening (5). Fundus images enable direct visualization of retinal vascular structures, optic disc, macula, and various pathological changes, providing rich imaging information for diagnosing multiple systemic diseases such as diabetes and hypertension, as well as ocular diseases including DR, glaucoma, and AMD (6). However, conventional manual interpretation relies heavily on ophthalmologists' expertise and clinical experience, which is not only time-consuming and labor-intensive–with an experienced ophthalmologist requiring an average of 2-5 minutes to interpret a single fundus image–but also subject to considerable inter-observer variability. More critically, in resource-limited regions, the severe shortage of ophthalmic specialists makes it difficult to meet the demands of large-scale screening (7). With the rapid advancement of deep learning technologies, automated fundus image analysis systems based on computer-aided diagnosis (CAD) have emerged, offering a novel technological pathway to address this clinical challenge (8, 9).

In the early stages of applying deep learning techniques to fundus image analysis, traditional machine learning methods dominated the field. These approaches typically relied on manually designed feature extraction operators, such as Local Binary Patterns (LBP) (10), Scale-Invariant Feature Transform (SIFT) (11), and Histogram of Oriented Gradients (HoG) (12), combined with classifiers including Support Vector Machines (SVM) (13), decision trees (14), or Artificial Neural Networks (ANN) (15) to achieve disease recognition. For instance, Chen et al. (16) proposed a hierarchical LBP feature representation method for diagnosing Branch Retinal Vein Occlusion (BRVO), achieving a classification accuracy of 82.3% on a proprietary dataset. Morales et al. (17) employed LBP features to extract texture characteristics from hard exudate regions and combined them with an SVM classifier to detect Diabetic Macular Edema (DME), attaining a sensitivity of 87.5%. For DR severity grading, Berbar (18) presented an automated method that required no lesion segmentation, utilizing image preprocessing and LBP feature extraction to train an SVM classifier that categorized DR severity into three levels with an accuracy of 89.7%. Although these methods achieved certain successes in specific tasks, the design of handcrafted features heavily depended on domain experts' prior knowledge and struggled to address the complex and variable pathological manifestations and illumination conditions in fundus images, resulting in limited model generalization capability (19).

In recent years, the emergence of deep learning models such as Convolutional Neural Networks (CNN) has fundamentally transformed the paradigm of fundus image analysis. Unlike traditional methods, deep learning models can automatically learn hierarchical feature representations from raw pixel data without requiring manual feature design (20). Since Google's team (21) first applied deep learning to DR screening in 2016 and achieved diagnostic performance comparable to ophthalmologists with an AUC of 0.99, numerous studies have subsequently proposed CNN-based fundus disease classification models. For example, Yaqoob et al. employed fine-tuned ResNet-50 as a feature extractor combined with random forest for classification, conducting DR detection and grading on EyePACS and Messidor-2, achieving 96% accuracy on the Messidor-2 binary classification task (22); Lin and Wu revised ResNet-50 with visualization guidance and preprocessing strategies, systematically analyzing the impact of key components on grading performance on EyePACS and reporting improved stability and generalization (23). In terms of network architectures, ResNet proposed by He et al. (24) alleviated gradient problems in deep networks through residual connections, while DenseNet proposed by Huang et al. (25) achieved feature reuse through dense connections, with these classic architectures being widely applied to fundus image analysis and yielding remarkable results. In the DenseNet direction, Kobat et al. utilized pre-trained DenseNet combined with horizontal and vertical patch partitioning to obtain robust DR grading results on APTOS 2019 and proprietary datasets (26); in the EfficientNet direction, Sivaz and Aykut combined the EfficientNet backbone with ML-Decoder classification head for ODIR-5K multi-label tasks, reporting Kappa of 68.96%, F1 of 92.48%, and AUC of 94.80%, outperforming various contemporary methods (27). Recently, Vision Transformers have also begun to be applied to fundus image analysis. For instance, Wang et al. proposed a novel Vision Transformer architecture for multi-label retinal disease classification, achieving superior performance compared to traditional CNNs on the ODIR-2019 dataset, with Kappa of 0.645, F1-score of 0.919, and AUC of 0.938 (28). These models have achieved diagnostic performance comparable to or even superior to human experts on multiple public datasets, demonstrating tremendous clinical application potential.

However, most existing deep learning methods primarily focus on single tasks, such as vessel segmentation or disease classification, failing to fully exploit the intrinsic relationships among different visual tasks in fundus images. Furthermore, as deep models are typically regarded as “black-box” systems, their decision-making processes lack transparency, which limits clinicians' trust and acceptance of AI systems (29). Although complete intrinsic interpretability remains an open challenge, *post-hoc* visualization techniques such as Grad-CAM can provide partial transparency by highlighting image regions influential to model predictions.

Notably, morphological alterations in the retinal vascular system constitute a common feature and early indicator of numerous fundal diseases. In DR, lesions such as microaneurysms, neovascularization, and hemorrhages directly reflect vascular structural abnormalities; in hypertensive retinopathy, arteriosclerosis and vascular narrowing serve as important diagnostic criteria; in glaucoma patients, peripapillary vascular changes are closely associated with disease progression (30). Therefore, accurate retinal vessel segmentation not only holds significant clinical value in itself but also provides crucial anatomical prior knowledge for disease diagnosis. In the field of vessel segmentation, numerous deep learning-based methods have been proposed. For example, the U-Net architecture proposed by Ronneberger et al. (31) achieved precise medical image segmentation through an encoder-decoder structure with skip connections, becoming a classic approach for retinal vessel segmentation. Building upon this foundation, Li et al. proposed Dense U-Net for retinal vessel segmentation, achieving an AUC of 0.9738, accuracy of 0.9698, sensitivity of 0.7931, and specificity of 0.989 on the DRIVE dataset (32). Additionally, Yue et al. proposed the SRV-GAN method based on generative adversarial networks, attaining an AUC of 0.9786, accuracy of 95.59%, sensitivity of 82.88%, and specificity of 97.45% on the DRIVE dataset (33). More recently, attention-based architectures have further advanced vessel segmentation performance. Ma et al. proposed VasCA-Net, which introduces a vascular channel attention mechanism to improve sensitivity to thin and low-contrast vessel structures (34). Li et al. proposed APU-Net, which employs dynamic feature fusion combined with a pyramid cross-attention mechanism to achieve fine-grained segmentation of complex vascular and tubular structures (35). These developments highlight the growing emphasis on attention-based feature modulation in medical image segmentation, a principle that V-MNet extends into the multi-task learning paradigm through its vessel-guided classification mechanism.

Although vessel segmentation and disease classification are closely related in clinical practice, existing studies typically treat them as independent tasks, constructing separate single-task models (36), failing to effectively leverage the synergistic information between the two tasks. Some studies have attempted to employ multi-task learning paradigms; for instance, Chai et al. (37) proposed a deep learning model combining frequent pattern mining modules and adversarial autoencoders for multi-label fundus image classification. However, existing multi-task learning methods mostly simply share low-level features, lacking explicit inter-task information transfer mechanisms. Particularly, no research has utilized the vessel segmentation task as prior guidance for disease classification, namely leveraging vascular structural information to guide and enhance disease diagnosis. This task isolation strategy not only limits further performance improvements but also fails to adequately reflect the holistic and systematic nature of clinical diagnosis.

Based on the above analysis, current deep learning-based fundus image analysis methods primarily face the following four key challenges: (1) Task independence issue: Vessel segmentation and disease classification are treated as mutually independent tasks, lacking a unified learning framework to exploit their intrinsic relationships and complementary information. Single-task models cannot leverage vascular structure as an important anatomical prior to guide disease diagnosis, resulting in insufficient information utilization. (2) Limited visual transparency: Most existing deep learning models lack effective visualization mechanisms, making it difficult for clinicians to understand which image regions influence model decisions. This opacity hinders clinical adoption. While full intrinsic interpretability remains an active research challenge, gradient-based visualization techniques such as Grad-CAM offer a practical pathway to partial transparency by highlighting spatially relevant regions (38). (3) Insufficient feature utilization: Although vascular lesions constitute core pathological features of numerous fundal diseases, existing classification models do not explicitly utilize vascular structural information to enhance disease diagnostic capabilities. This results in models lacking specificity and robustness when extracting pathological features. (4) Suboptimal performance and overfitting risk: Single-task learning is prone to falling into local optima and easily suffers from overfitting on small-sample datasets (39). The absence of inter-task regularization effects and shared representation learning limits the model's generalization capability and diagnostic accuracy.

To address the aforementioned challenges, this study proposes a novel multi-task deep learning framework–V-MNet (Vessel-guided Multi-task Network)–aiming to simultaneously achieve retinal vessel segmentation and multi-disease classification through a unified end-to-end learning paradigm while enhancing model interpretability. The core innovation of V-MNet lies in learning cross-task universal feature representations through a shared encoder, utilizing structural prior knowledge provided by the vessel segmentation task to guide the disease classification task, and generating visual explanations through Gradient-weighted Class Activation Mapping (Grad-CAM) (40) technology, enabling clinicians to intuitively understand the model's decision-making process. The main contributions of this study can be summarized in the following four aspects: (1) Unified multi-task learning framework: For the first time, retinal vessel segmentation and multi-disease classification are integrated into a unified deep learning architecture, achieving mutual promotion and performance enhancement of both tasks through inter-task collaborative learning and information sharing. (2) Vessel-guided feature fusion mechanism: Innovatively designed vessel-guided module that explicitly integrates structural information extracted by the vessel segmentation branch into the disease classification branch, enabling the model to more precisely localize and identify pathological regions associated with vascular lesions. (3) Visual transparency support: Integration of Grad-CAM visualization technology to generate class activation maps for each disease category, providing *post-hoc* visual evidence of the spatially relevant regions driving model predictions, thereby supporting clinician review and building trust in AI-assisted diagnosis. (4) Superior experimental performance: Comprehensive validation on four public datasets–RFMiD, ODIR-5K, DRIVE, and EyePACS-light-v2–with experimental results demonstrating that V-MNet achieves a Dice coefficient of 0.831 and AUC of 0.985 for vessel segmentation tasks, and an average AUC of 0.978 with F1-score of 0.935 for multi-disease classification tasks, significantly outperforming single-task baseline models and existing state-of-the-art methods.

The remainder of this paper is organized as follows: Section 2 elaborates on the overall architecture of the V-MNet framework, the design principles of each functional module, and the training strategy; Section 3 introduces the experimental setup, evaluation metrics, and baseline methods; Section 4 presents detailed experimental results, ablation analyses, and visualization cases; Section 5 provides an in-depth discussion of the experimental results, analyzing the advantages of multi-task learning, the role of the vessel-guided mechanism, the clinical value of interpretability, and research limitations; Section 6 summarizes the entire work and outlines future research directions.

## 2 Materials and methods

### 2.1 Overall framework architecture

To address the issues of task independence, inadequate interpretability, and insufficient feature utilization in existing fundus image analysis methods, this study proposes V-MNet (Vessel-guided Multi-task Network), a multi-task deep learning framework. The framework simultaneously achieves retinal vessel segmentation and multi-disease classification through a unified end-to-end learning paradigm while leveraging vascular structural priors to enhance disease diagnostic capabilities.

**Figure 1:**
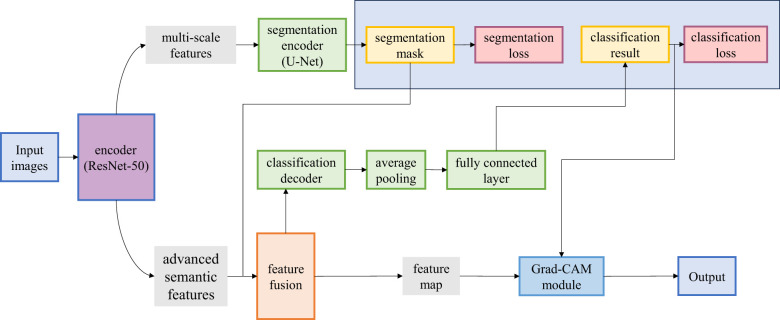
V-MNet architecture showing vessel-guided multi-task learning with explicit feature flow paths between segmentation and classification branches.

As illustrated in Figure 1, V-MNet adopts a “shared encoder-dual decoder” architecture comprising four core modules: The diagram explicitly shows the feature flow paths, including how the vessel segmentation mask *M*_*seg*_ is downsampled and fused with encoder features *F*_4_ to generate the vessel-guided feature *f*_*vessel*_, which is then concatenated with the global feature *f*_*global*_ to form the enhanced classification feature *f*_*enhanced*_. the Shared Encoder extracts multi-scale feature representations; the Segmentation Decoder generates vessel segmentation masks through a U-Net-style upsampling structure; the Classification Decoder accomplishes disease classification through vessel-guided feature fusion; and the Grad-CAM module produces class activation maps to provide interpretability support.

Data flow and task synergy mechanism: Given an input fundus image **I**∈ℝ^*H*×*W*×3^, where *H* and *W* represent the height and width of the image respectively, the image is first preprocessed and then fed into the shared encoder. The encoder employs pre-trained ResNet-50 as the backbone network and progressively extracts a multi-scale feature pyramid {*F*_1_, *F*_2_, *F*_3_, *F*_4_, *F*_5_} through residual connections, where $F_{i}\in {\mathbb{R}}^{H_{i}\times W_{i}\times C_{i}}$ denotes the feature map at the *i*-th layer.

Following the encoder, the feature stream is divided into two parallel task-specific branches. The segmentation decoder adopts a U-Net-style architecture and fuses high-level semantic features with shallow spatial detail features through progressive upsampling and skip connection mechanisms, ultimately generating a vessel segmentation mask $M_{seg}\in {\left\{0,1\right\}}^{H\times W}$ at the same resolution as the input image, where 1 indicates vessel pixels and 0 indicates background pixels. Concurrently, the classification decoder first compresses the high-level semantic feature F5 output by the encoder into a global descriptor vector $f^{global}\in {\mathbb{R}}^{C_{5}}$ through Global Average Pooling (GAP).

Vessel-guided feature fusion mechanism: The core innovation of V-MNet lies in explicitly utilizing the structural priors provided by the vessel segmentation task to guide disease classification. Specifically, the vessel mask **M**_*seg*_ generated by the segmentation decoder is downsampled through the Feature Fusion Module and then interacts with the semantic feature F5 via channel-wise and spatial-wise attention mechanisms, generating an enhanced feature *f*_*enhanced*_. This design enables the classification network to focus more precisely on key regions associated with vascular lesions, thereby improving the accuracy of disease diagnosis.

Multi-label classification and interpretability output: The enhanced feature *f*_*enhanced*_ is mapped to the multi-label output space through a two-layer fully connected network and generates prediction probabilities for each disease category $y=\left[y_{1},y_{2},...,y_{C}\right]\in {\left[0,1\right]}^{C}$ using the Sigmoid activation function, where C represents the total number of disease categories. To enhance the clinical interpretability of the model, V-MNet integrates the Grad-CAM module [3], which computes the gradient of the classification loss with respect to the feature maps to generate class activation maps ${CAM}_{c}\in {\mathbb{R}}^{H\times W}$ for each disease category, clearly identifying the lesion regions attended by the model.

End-to-end joint optimization: V-MNet employs a multi-task loss function for end-to-end training, where the total loss ${ℒ}_{total}$ is defined as the weighted sum of the segmentation loss ${ℒ}_{seg}$ and the classification loss ${ℒ}_{cls}$:


$$\begin{array}{l} {ℒ}_{total}=\lambda_{seg}{ℒ}_{seg}+\lambda_{cls}{ℒ}_{cls} \end{array}$$ (1)

where λ_*seg*_ and λ_*cls*_ are weight hyperparameters. The segmentation loss adopts Dice Loss to address the class imbalance problem between foreground and background, while the classification loss employs Binary Cross-Entropy to accommodate the multi-label classification task. Through joint optimization, a regularization effect and information complementarity are established between the two tasks, enabling the shared encoder to learn universal feature representations beneficial to both tasks while avoiding the overfitting problem prone to single-task models.

### 2.2 Datasets and data preprocessing

To comprehensively evaluate the performance and generalization capability of V-MNet, this study employs four public fundus image datasets: RFMiD, ODIR-5K, DRIVE, and EyePACS-light-v2. These datasets exhibit distinctive characteristics in terms of scale, disease types, annotation quality, and image sources, enabling thorough testing of the model's robustness across diverse clinical scenarios.

**Figure 2:**
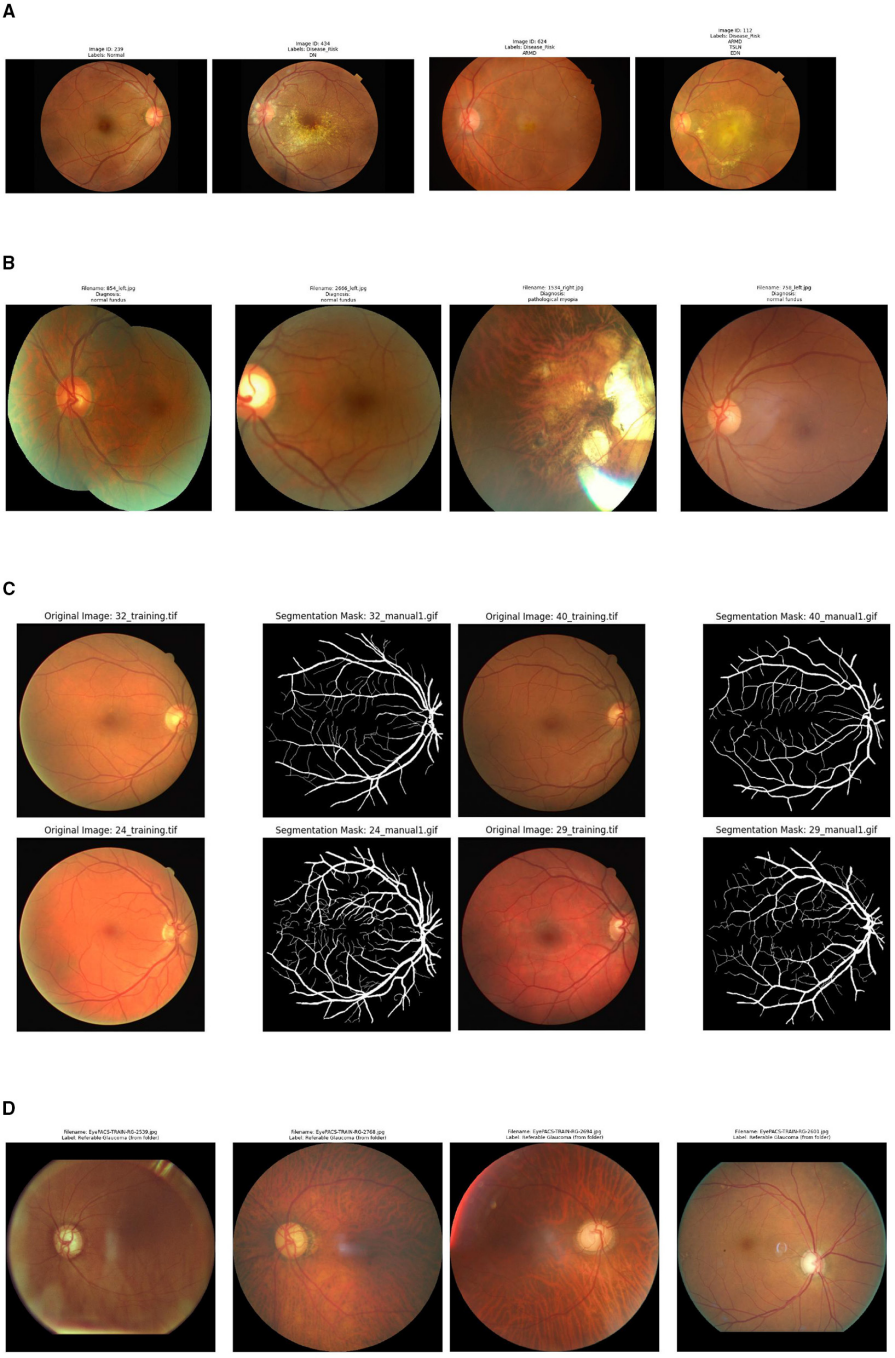
Sample dataset display. **(A)** RFMiD dataset sample. **(B)** ODIR-5K dataset sample. **(C)** DRIVE dataset sample. **(D)** EyePACS-light-v2 dataset sample.

As illustrated in Figure 2A, RFMiD (Retinal Fundus Multi-Disease Image Dataset) (41) is a dataset specifically designed for multi-disease, multi-label classification, released by Shanggong Medical Technology Co. in India. The dataset comprises 3200 color fundus images covering 46 disease labels including DR, AMD, glaucoma, and cataracts, with over 23% being multi-label samples. Images were acquired using three different brands of fundus cameras (TOPCON 3D OCT-2000, Kowa VX-10α, and TOPCON TRC-NW300), with resolutions ranging from 1536 × 2048 to 2848 × 4288. All annotations were independently completed by at least two ophthalmologists, with disagreements resolved by a third expert to ensure high-quality labeling. In this study, RFMiD is primarily utilized for training and validating multi-label disease classification tasks.

As illustrated in Figure 2B, ODIR-5K (Ocular Disease Intelligent Recognition) (42) is a large-scale database released for the 2019 ODIR competition, containing bilateral fundus photographs from 5,000 patients across multiple hospitals in China. The dataset is categorized into eight disease classes: Normal (N), Diabetic Retinopathy (D), Glaucoma (G), Cataract (C), AMD (A), Hypertensive Retinopathy (H), Myopia (M), and Other Abnormalities (O). Due to acquisition from different medical institutions using multiple camera brands including Canon, Zeiss, and Kowa, the images exhibit substantial variations in resolution, color, and quality, making them suitable for testing cross-domain generalization capabilities. ODIR-5K provides patient-level annotations and detailed clinical metadata, and is primarily used for external validation in this study.

As illustrated in Figure 2C, DRIVE (Digital Retinal Images for Vessel Extraction) (43) is a classic benchmark dataset for retinal vessel segmentation, collected by the University Medical Center Utrecht in the Netherlands. The dataset contains 40 color fundus images with a resolution of 565 × 584, equally divided into 20 training and 20 test images. Each image is accompanied by vessel masks independently annotated by two experts, with the test set providing two sets of annotations to assess inter-observer consistency. Despite its relatively small scale, DRIVE has become the gold standard for evaluating vessel segmentation algorithms due to its high-quality manual annotations and widespread academic recognition. In this study, DRIVE is specifically employed to validate the performance of V-MNet's vessel segmentation branch.

As illustrated in Figure 2D, EyePACS-light-v2 (44) is a preprocessed glaucoma classification subset derived from the Kaggle DR competition dataset, constructed through Rotterdam EyePACS AIROGS screening. This dataset addresses issues such as excessively large image sizes and cumbersome preprocessing in the original EyePACS dataset by applying uniform preprocessing to all images, including black border removal and standardization to 512 × 512 pixels, making them directly usable for model training. The dataset comprises three classes: Referable-glaucoma, Non-referable, and Ungradable, and provides predefined train/val/test splits along with detailed metadata. In this study, EyePACS-light-v2 serves as an external test set to evaluate cross-domain generalization performance for glaucoma detection.

Table 1 provides a detailed summary of the core characteristics of the four datasets.

**Table 1:** Summary of core dataset characteristics.

Dataset	Task	Scale and subjects	Labels	Primary use
RFMiD	Multi-disease multi-label classification	3,200 color fundus images from 3 camera types	46 pathological labels; ≥23% multi-label samples	Training/validation for multi-label classification; long-tail/multi-domain generalization
ODIR-5K	Multi-disease multi-label classification	5,000 patients, one image per eye	8 classes: N, D, G, C, AMD, H, M, O	Training/validation for cross-domain generalization and multi-label classification
DRIVE	Vessel segmentation	40 color fundus images	Segmentation masks	External testing for vessel segmentation generalization
EyePACS-light-v2	Glaucoma binary classification	Balanced standardized subset from AIROGS	Referable-glaucoma / Non-referable / Ungradable	External testing for cross-dataset classification robustness and domain shift

To ensure training effectiveness and experimental comparability, this study applies uniform preprocessing to all datasets: all images are resized to 224 × 224 pixels to accommodate ResNet-50 input requirements, and pixel values are normalized to the [0,1] range. For vessel segmentation tasks, DRIVE training images undergo additional preprocessing including green channel extraction, CLAHE histogram equalization, and Gamma correction to enhance vessel contrast. To mitigate data imbalance and enhance generalization capability, various data augmentation techniques are employed during training, including random horizontal/vertical flipping, rotation (±15°), and brightness/contrast adjustment.

Regarding data splitting: RFMiD and ODIR-5K are randomly divided into 70% training, 20% testing, and 10% validation sets, ensuring consistent disease class distribution across subsets; DRIVE adopts the official 20/20 training/test split; EyePACS-light-v2 utilizes the officially predefined train/val/test splits. All splits are fixed prior to experimentation to ensure reproducibility. To address the severe class imbalance in the RFMiD dataset—where certain rare disease categories contain fewer than 10 training samples—this study adopts a dynamic class-weighted loss strategy during training. Specifically, for each disease category *c*, the positive class weight is computed as the ratio of negative to positive samples: *w*_*c*_ = (*N*−*n*_*c*_)/*n*_*c*_, where *N* is the total number of training samples and *n*_*c*_ is the number of positive samples for category *c*. These weights are applied to the Binary Cross-Entropy loss function to penalize misclassification of minority categories more heavily. In addition, multi-label stratified sampling is employed during dataset splitting to maintain consistent label distribution across training, validation, and test subsets, ensuring that rare categories are represented proportionally in each split.

In summary, the four datasets employed in this study form excellent complementarity in terms of task types, data scale, disease coverage, and data source diversity, enabling comprehensive and fair evaluation of the V-MNet framework's performance on vessel segmentation and multi-disease classification tasks, while validating its application potential in clinical practice.

### 2.3 Network architecture

The network architecture of V-MNet achieves synergistic optimization of vessel segmentation and disease classification through carefully designed modular components. This section elaborates on the structural design of each functional module.

#### 2.3.1 Shared encoder

The shared encoder employs ResNet-50 (45) pre-trained on ImageNet as the backbone network. ResNet-50 progressively extracts a multi-scale feature pyramid {*F*_1_, *F*_2_, *F*_3_, *F*_4_} through four residual stages, where *F*_*i*_ represents the feature map output by the *i*-th stage. The residual connection mechanism effectively mitigates the gradient vanishing problem, enabling the network to learn deep features while maintaining training stability. Through ImageNet pre-training, the encoder acquires universal visual prior knowledge, establishing a foundation for small-sample medical image analysis.

#### 2.3.2 Segmentation decoder

The segmentation decoder adopts a U-Net (46)-style upsampling architecture to achieve pixel-level vessel segmentation. The decoder consists of four upsampling blocks, each comprising a transposed convolutional layer (to expand feature map dimensions), skip connections (to fuse features from corresponding encoder layers), and two 3 × 3 convolutional layers (followed by BatchNorm and ReLU). Skip connections effectively combine high-level semantic information with low-level spatial details, enabling the segmentation network to perform both coarse localization and precise pixel-level prediction. Finally, a 1 × 1 convolutional layer followed by a Sigmoid activation function generates the vessel segmentation probability map $M_{seg}\in {\left[0,1\right]}^{H\times W}$.

#### 2.3.3 Classification decoder and vessel-guided mechanism

The classification decoder integrates global semantic features with vascular structural priors, constituting the core innovative module of V-MNet. The high-level feature *F*_4_ output by the encoder is first compressed into a global feature vector *f*_*global*_ through Global Average Pooling (GAP), capturing the global pathological patterns of the image.

The vessel-guided spatial attention mechanism represents a key innovation of V-MNet. Formally, the vessel-guided feature *f*_*vessel*_ is computed as:


$$\begin{array}{l} f_{vessel}=F_{4}⊙\text{Downsample}\left(M_{seg}\right) \end{array}$$ (2)

where ⊙ denotes element-wise multiplication, $F_{4}\in {\mathbb{R}}^{H_{4}\times W_{4}\times C_{4}}$ is the high-level feature map from the encoder, and Downsample(·) spatially resizes the segmentation mask $M_{seg}\in {\left[0,1\right]}^{H\times W}$ to match the spatial dimensions of *F*_4_. This operation enhances feature responses related to vessels in *F*_4_ through element-wise multiplication, generating the vessel-guided feature *f*_*vessel*_. The global feature and vessel-guided feature are fused through concatenation to form *f*_*fused*_, which simultaneously encodes global pathological patterns and vascular structural information. The fused feature is mapped to the multi-label output space through a two-layer fully connected network (with Dropout in the intermediate layer to prevent overfitting), generating the prediction probability vector **y**∈[0, 1]^*C*^ using the Sigmoid activation function.

#### 2.3.4 Grad-CAM explainability module

V-MNet integrates the Grad-CAM (Gradient-weighted Class Activation Mapping) module to enhance clinical interpretability. For a target disease category *c*, Grad-CAM computes the gradient of the classification score with respect to the feature map *F*_4_, generating channel importance weights, which are subsequently weighted and combined followed by ReLU activation to produce class activation maps. After upsampling to the original image size, this heatmap clearly identifies the key lesion regions attended by the model, providing transparent decision-making evidence for clinicians.

### 2.4 Loss function and training strategy

#### 2.4.1 Multi-task loss function

V-MNet employs a multi-task learning paradigm, where the total loss function is a weighted combination of segmentation loss and classification loss:


$$\begin{array}{l} {ℒ}_{total}=\lambda_{seg}{ℒ}_{seg}+\lambda_{cls}{ℒ}_{cls} \end{array}$$ (3)

Through grid search, we set λ_*seg*_ = 0.5, λ_*cls*_ = 0.5. The segmentation loss adopts Dice Loss to address class imbalance:


$$\begin{array}{l} {ℒ}_{seg}=1-\frac{2\underset{i}{\sum }p_{i}g_{i}+\epsilon }{\underset{i}{\sum }p_{i}+\underset{i}{\sum }g_{i}+\epsilon } \end{array}$$ (4)

where *p*_*i*_ represents the predicted pixel value, *g*_*i*_ denotes the ground truth label, and ϵ = 1 is a smoothing term. The classification loss employs Binary Cross-Entropy (BCE) to accommodate multi-label scenarios:


$$\begin{array}{l} {ℒ}_{cls}=-\frac{1}{C}\overset{C}{\underset{c=1}{\sum }}\left[t_{c}\log\left(y_{c}\right)+\left(1-t_{c}\right)\log\left(1-y_{c}\right)\right] \end{array}$$ (5)

#### 2.4.2 Training strategy

V-MNet adopts a two-stage training strategy. In the first stage, encoder parameters are frozen, and only the decoders are trained for 20 epochs; in the second stage, the encoder is unfrozen for end-to-end fine-tuning over 80 epochs. Training employs the Adam optimizer with an initial learning rate of 1 × 10^−4^, dynamically adjusted to a minimum value of 1 × 10^−6^ using a cosine annealing schedule. The batch size is set to 16, with weight decay of 10^−4^. During training, data augmentation techniques including random horizontal/vertical flipping, rotation (±15°), and brightness/contrast adjustment are applied. Early stopping is employed to monitor validation set AUC, terminating training if no improvement is observed for 15 consecutive epochs. All experiments are implemented using PyTorch 1.12.0 on a workstation equipped with NVIDIA TITAN V GPUs.

## 3 Experimental setup

### 3.1 Evaluation metrics

To comprehensively evaluate the performance of V-MNet on both vessel segmentation and multi-disease classification tasks, this study employs a targeted evaluation metric system. Considering the differences in task characteristics, corresponding evaluation metrics are designed for each task.

#### 3.1.1 Vessel segmentation task metrics

Vessel segmentation, as a pixel-level binary classification task, employs the following three core metrics to evaluate segmentation quality.

##### 3.1.1.1 Dice coefficient

The Dice coefficient measures the degree of overlap between the predicted mask and the ground truth annotation, defined as:


$$\begin{array}{l} \text{Dice}=\frac{2\times |P\cap G|}{\left|P|+|G\right|}=\frac{2\times \text{TP}}{2\times \text{TP}+\text{FP}+\text{FN}} \end{array}$$ (6)

where *P* represents the set of predicted vessel pixels, *G* denotes the set of ground truth vessel pixels, and TP, FP, and FN represent the numbers of true positive, false positive, and false negative pixels, respectively. The Dice coefficient ranges from [0, 1], with values closer to 1 indicating greater similarity between segmentation results and ground truth annotations. This metric is robust to class imbalance problems and is particularly suitable for medical image segmentation tasks where foreground pixels (vessels) constitute a small proportion. The Dice coefficient is a standard metric for evaluating vessel segmentation algorithms and is widely used for performance comparison on benchmark datasets such as DRIVE.

##### 3.1.1.2 Area under the curve (AUC)

AUC evaluates the comprehensive performance of a classifier across different thresholds by calculating the area under the ROC curve:


$$\begin{array}{l} \text{AUC}=\overset{1}{\underset{0}{\int }}\text{TPR}\left(t\right)d\left[\text{FPR}\left(t\right)\right] \end{array}$$ (7)

where TPR (True Positive Rate, i.e., sensitivity) $=\frac{\text{TP}}{\text{TP}+\text{FN}}$, and FPR (False Positive Rate) $=\frac{\text{FP}}{\text{FP}+\text{TN}}$. AUC ranges from [0, 1], with larger values indicating better classification performance. AUC is independent of classification thresholds and can comprehensively reflect model performance across various operating points, making it an important metric for evaluating medical image segmentation models (32).

##### 3.1.1.3 Accuracy

Accuracy measures the proportion of correctly classified pixels:


$$\begin{array}{l} \text{Accuracy}=\frac{\text{TP}+\text{TN}}{\text{TP}+\text{TN}+\text{FP}+\text{FN}} \end{array}$$ (8)

where TN represents the number of true negative pixels. Although accuracy may be misleading in class imbalance problems (background pixels far outnumber vessel pixels), combined with Dice coefficient and AUC metrics, it provides a more comprehensive performance evaluation. In the official evaluation of the DRIVE dataset, accuracy is also one of the commonly used comparison metrics.

#### 3.1.2 Disease classification task metrics

Multi-label disease classification tasks require evaluation of the model's diagnostic capability for each disease category. This study employs the following six metrics.

##### 3.1.2.1 Precision

Precision measures the proportion of samples predicted as positive that are truly positive. For disease category *c*, it is defined as:


$$\begin{array}{l} {\text{Precision}}_{c}=\frac{{\text{TP}}_{c}}{{\text{TP}}_{c}+{\text{FP}}_{c}} \end{array}$$ (9)

where TP_*c*_ represents the number of true positive samples for category *c*, and FP_*c*_ denotes the number of false positive samples. High precision means lower misdiagnosis rates, which in clinical applications can reduce unnecessary further examinations and treatments, lower healthcare costs, and alleviate patients' psychological burden (4). This study reports the average precision across all disease categories.

##### 3.1.2.2 Recall (sensitivity)

Recall, also known as sensitivity, measures the proportion of true positive samples correctly identified by the model. For disease category *c*, it is defined as:


$$\begin{array}{l} {\text{Recall}}_{c}=\frac{{\text{TP}}_{c}}{{\text{TP}}_{c}+{\text{FN}}_{c}} \end{array}$$ (10)

where FN_*c*_ represents the number of false negative samples for category *c*. High recall means lower missed diagnosis rates, which is crucial for early screening and prevention of irreversible vision loss (5). In disease screening scenarios, recall is often more important than precision, as missed diagnoses may cause patients to miss optimal treatment opportunities.

##### 3.1.2.3 F1-score

The F1-score is the harmonic mean of precision and recall, comprehensively reflecting the model's diagnostic performance:


$$\begin{array}{l} {\text{F1-Score}}_{c}=\frac{2\times {\text{Precision}}_{c}\times {\text{Recall}}_{c}}{{\text{Precision}}_{c}+{\text{Recall}}_{c}}=\frac{2\times {\text{TP}}_{c}}{2\times {\text{TP}}_{c}+{\text{FP}}_{c}+{\text{FN}}_{c}} \end{array}$$ (11)

The F1-score balances the weights of precision and recall, avoiding biases that may arise from single metrics. This metric ranges from [0, 1], with values closer to 1 indicating better model performance. The F1-score is particularly suitable for evaluating overall performance across categories in multi-label classification (41). This study reports F1-scores for individual disease categories and their average.

##### 3.1.2.4 Specificity

Specificity measures the model's ability to correctly identify negative samples. For disease category *c*, it is defined as:


$$\begin{array}{l} {\text{Specificity}}_{c}=\frac{{\text{TN}}_{c}}{{\text{TN}}_{c}+{\text{FP}}_{c}} \end{array}$$ (12)

where TN_*c*_ represents the number of true negative samples for category *c*. High specificity means the model can accurately exclude healthy individuals, avoiding overdiagnosis and unnecessary medical interventions. In large-scale population screening, higher specificity can effectively reduce false positive rates and minimize waste of medical resources (6).

##### 3.1.2.5 Accuracy

For multi-label classification tasks, accuracy measures the proportion of samples with completely correct classifications:


$$\begin{array}{l} \text{Accuracy}=\frac{1}{N}\overset{N}{\underset{i=1}{\sum }}⊮\left[y_{i}={\hat{y}}_{i}\right] \end{array}$$ (13)

where *N* is the total number of samples, **y**_*i*_ is the true label vector for the *i*-th sample, ${\hat{y}}_{i}$ is the predicted label vector, and ⊮[·] is the indicator function (taking 1 when the condition is true, 0 otherwise). This metric reflects the model's overall accuracy across all disease categories.

##### 3.1.2.6 Mean AUC

For multi-label classification tasks, the AUC value for each disease category is first calculated, then the arithmetic mean is computed:


$$\begin{array}{l} \text{Mean AUC}=\frac{1}{C}\overset{C}{\underset{c=1}{\sum }}{\text{AUC}}_{c} \end{array}$$ (14)

where *C* is the total number of disease categories, and AUC_*c*_ is the area under the ROC curve for category *c*. Mean AUC comprehensively reflects the model's overall classification performance across all disease categories and is the most important comprehensive evaluation metric for multi-label medical image classification tasks. AUC is independent of classification thresholds and sample imbalance, enabling objective evaluation of the model's discriminative capability.

In addition to segmentation and classification performance metrics, Table 2 summarizes the computational profiles of V-MNet and all baseline methods, measured under identical hardware conditions on a single NVIDIA TITAN V GPU with batch size of 1. V-MNet contains approximately 32.5 million parameters with a total computational cost of 8.7 GFLOPs per inference and an average inference time of 18.3 milliseconds per image, corresponding to a throughput of approximately 54 images per second. Compared to the Dfex-BeeHive Model, which incurs the highest computational overhead among all evaluated methods (42.1M parameters, 11.8 GFLOPs, 39.4 ms/image), V-MNet reduces parameter count by 22.8%, FLOPs by 26.3%, and inference latency by 53.6%, while achieving consistently superior performance on both tasks. Relative to single-task baselines such as CNN-RNN, V-MNet's dual-decoder multi-task architecture introduces only modest additional computation (+3.8M parameters, +2.6 GFLOPs) while delivering meaningful gains across all evaluation metrics. Among multi-task baselines, V-MNet achieves the lowest inference latency (18.3 ms) while outperforming both MTL-Fundus and MTDCN on segmentation and classification benchmarks. These results indicate that V-MNet achieves a favorable accuracy–efficiency trade-off suitable for GPU-based batch screening workflows, though deployment on mobile or edge devices would require further model compression, as discussed in Section 5.5.

**Table 2:** Computational efficiency comparison of all methods.

Method	Parameters (M)	FLOPs (G)	Inference time (ms/image)
CNN-RNN	28.7	6.1	26.4
Dfex-BeeHive Model	42.1	11.8	39.4
Vision Track	36.3	9.4	32.7
MTL-Fundus	30.6	8.1	22.1
MTDCN	34.2	9.3	28.5
V-MNet (ours)	32.5	8.7	18.3

In summary, this study employs three metrics (Dice coefficient, AUC, and accuracy) for vessel segmentation tasks, and six metrics (precision, recall, F1-score, specificity, accuracy, and mean AUC) for disease classification tasks, constructing a comprehensive evaluation system. These metrics quantify model performance from different perspectives, focusing on both pixel-level segmentation accuracy and clinical diagnostic accuracy, sensitivity, and specificity, ensuring the comprehensiveness and clinical relevance of evaluation results.

### 3.2 Dataset splitting strategy

To ensure the reliability and reproducibility of experimental results, this study adopts strict splitting strategies for the four datasets.

#### 3.2.1 RFMiD dataset

Random stratified sampling is employed to divide the 3,200 images into training (70%, 2,240 images), test (20%, 640 images), and validation (10%, 320 images) sets. Stratified sampling ensures that the distribution proportions of the 46 disease categories remain consistent across the three subsets, avoiding the impact of class imbalance on model training and evaluation. Considering that over 23% of images have multi-label characteristics, particular attention is paid to maintaining balanced distribution of multi-label samples across subsets during splitting. All experiments use the same random seed (seed = 42) to ensure reproducibility of the splits.

#### 3.2.2 ODIR-5K dataset

Since the original dataset contains 10,000 images (bilateral images from 5,000 patients), this study similarly adopts a 70:20:10 split ratio, generating training (7,000 images), test (2,000 images), and validation (1,000 images) sets. Considering the potential physiological correlation and disease association between bilateral images of the same patient, the splitting ensures that left and right eye images from the same patient are assigned to the same subset, strictly avoiding data leakage issues. This strategy ensures that the model faces completely unseen patients during testing, more realistically reflecting generalization capability in clinical application scenarios.

#### 3.2.3 DRIVE dataset

The official standard splitting scheme is adopted, with 20 images for training and 20 images for testing. This splitting scheme has been widely used by the international academic community since the dataset's release in 2004 and serves as the gold standard for evaluating vessel segmentation algorithms. Adopting the official split facilitates fair and direct performance comparison with published literature. Due to DRIVE's small scale, data augmentation techniques are employed during training to expand training samples and prevent overfitting.

#### 3.2.4 EyePACS-light-v2 dataset

The officially predefined train/val/test split is adopted. This dataset, preprocessed and standardized (uniformly 512 × 512 pixels with black borders removed), is specifically used as an external independent test set to evaluate the model's cross-domain generalization capability on glaucoma detection tasks. This dataset does not participate in any training or hyperparameter tuning process of V-MNet, ensuring unbiased and objective evaluation results.

All dataset splitting information is fixed and saved as configuration files before the experiments begin, ensuring that subsequent ablation experiments, comparative experiments, and visualization analyses use exactly the same data subsets, guaranteeing experimental controllability and reproducibility.

### 3.3 Baseline methods

To comprehensively evaluate the performance advantages of V-MNet, this study selects three representative state-of-the-art methods as baselines for comparison. These methods have all achieved excellent performance in the field of fundus image analysis and can fully reflect the current state of the art. All baseline methods are trained on the same datasets (RFMiD and ODIR-5K) and evaluated on the same test sets (DRIVE and EyePACS-light-v2) to ensure fair comparison. It should be noted that the selected baselines, while representative, were not originally designed specifically for unified multi-task fundus analysis. To provide a more contemporary comparison, this study additionally includes two recent multi-task deep learning methods—MTL-Fundus (47) and MTDCN (48)—which have been published within the past three years and address related tasks in fundus image analysis. Due to the rapid development of the field, further comparisons with emerging methods will be incorporated in future work as reproducible implementations become publicly available.

#### 3.3.1 CNN-RNN

CNN-RNN is a hybrid architecture combining convolutional neural networks and recurrent neural networks, specifically designed to capture spatial and sequential features in fundus images. This method first uses CNN to extract local spatial features from images, then models long-range dependencies between features through RNN (typically LSTM or GRU). In vessel segmentation tasks, CNN-RNN can effectively capture vessel continuity and topological structure; in disease classification tasks, this architecture can integrate multi-scale pathological features for comprehensive judgment. CNN-RNN represents the application of sequence modeling techniques in medical image analysis and has demonstrated good performance on multiple fundus image datasets.

#### 3.3.2 Dfex-BeeHive model

The Dfex-BeeHive Model is a method based on deep feature extraction and swarm intelligence optimization. This model employs deep convolutional networks to extract discriminative features from images (Deep Feature Extraction) and combines the BeeHive optimization algorithm inspired by bee swarm behavior for feature selection and model optimization. The BeeHive algorithm can efficiently search for optimal feature subsets in high-dimensional feature spaces by simulating bee foraging and information sharing mechanisms, avoiding interference from redundant features. This method performs excellently in multi-label disease classification tasks and is particularly suitable for handling class imbalance and feature redundancy problems. The Dfex-BeeHive Model represents the technical approach of combining bio-inspired optimization algorithms with deep learning.

#### 3.3.3 Vision track

Vision Track is a method based on visual tracking and attention mechanisms, specifically designed for lesion localization and disease recognition tasks in fundus images. This method automatically identifies and localizes key anatomical structures (such as optic disc, macula, vessels) and pathological regions (such as exudates, hemorrhages, microaneurysms) through tracking algorithms, then uses attention mechanisms to focus on these key regions for classification. The core idea of Vision Track is to simulate the reading process of ophthalmologists by progressively focusing on important regions to improve diagnostic accuracy. This method has achieved competitive results on both vessel segmentation and disease classification tasks, representing the application of attention mechanisms and tracking techniques in medical image analysis.

For each baseline method, this study strictly follows the network architecture and key hyperparameter settings described in their original papers, only adjusting batch size and training epochs to accommodate the dataset scales of this study. All methods are trained until convergence to ensure they achieve optimal performance.

#### 3.3.4 MTL-Fundus

MTL-Fundus is a recent multi-task learning framework specifically designed for simultaneous retinal vessel segmentation and disease classification from fundus images. This method employs a shared convolutional backbone with dual task-specific decoders and introduces a cross-task feature interaction module that enables bidirectional information exchange between the segmentation and classification branches. By jointly training on vessel-annotated and disease-labeled datasets, MTL-Fundus demonstrates improved performance over single-task counterparts and serves as a strong contemporaneous multi-task baseline for fundus image analysis (47).

#### 3.3.5 MTDCN

MTDCN (Multi-Task Dual-path Convolutional Network) is a recently proposed framework for multi-label ophthalmic disease recognition that incorporates both local lesion-level and global image-level features through a dual-path architecture. The network leverages an attention-based feature aggregation mechanism to selectively emphasize disease-relevant regions while suppressing background noise. MTDCN has achieved competitive results on multiple public fundus datasets and represents the state of the art in attention-guided multi-task medical image analysis (48).

### 3.4 Ablation study design

To systematically verify the effectiveness and necessity of each key component in the V-MNet framework, this study designs three groups of comparative experiments. By progressively removing or modifying core modules, the marginal contribution of each component to final performance is analyzed. The experiments focus on evaluating two key factors: the transfer learning effect of the ImageNet pre-training strategy on medical imaging tasks, and the performance gain of the multi-task learning paradigm (integrating vessel segmentation and disease classification) compared to single-task methods.

**Experiment 1: Full V-MNet model**

This is the complete framework proposed in this paper, containing all functional modules. The ResNet-50 encoder is initialized with ImageNet pre-trained weights, fully leveraging universal visual feature representations learned from large-scale natural image datasets. The model simultaneously trains vessel segmentation and disease classification tasks, achieving feature reuse through a shared encoder. The segmentation decoder adopts a U-Net-style upsampling structure, progressively restoring spatial resolution through skip connections to generate pixel-level vessel segmentation masks. The classification decoder, based on encoder output features, integrates vessel-guided spatial attention information and generates multi-label disease predictions through global average pooling and fully connected layers. The loss function is a weighted combination: ${ℒ}_{total}=0.4{ℒ}_{seg}+0.6{ℒ}_{cls}$, where ${ℒ}_{seg}$ is the Dice loss and ${ℒ}_{cls}$ is the binary cross-entropy loss. Additionally, the model integrates a Grad-CAM interpretability module to generate class activation maps for each disease category. This configuration represents the optimal performance upper bound of V-MNet.

**Experiment 2: V-MNet without pre-training**

This configuration maintains the same network architecture, multi-task learning paradigm, vessel-guided mechanism, and loss function weights as the full model, with the only difference being the initialization strategy of the ResNet-50 encoder. The encoder uses Kaiming He initialization (also known as He initialization) to randomly initialize all convolutional layer weights without using any pre-trained weights. This configuration verifies the critical role of transfer learning strategies in medical image analysis. Medical image datasets are typically limited in scale (approximately 8,000 images in the training set of this study), making deep networks trained from scratch prone to overfitting and local optima. By comparing the performance gap between Experiment 1 and Experiment 2, the performance improvement brought by ImageNet pre-trained weights to vessel segmentation and disease classification tasks can be quantified, evaluating the transferability of universal visual features such as edges, textures, and shapes learned from large-scale natural image data to the medical imaging domain.

**Experiment 3: single-task baseline models**

This experiment includes two independent single-task configurations to verify the overall gain effect of multi-task learning. The first configuration is a single-task vessel segmentation model (Segmentation Only), which completely removes the disease classification branch and its related modules, degenerating into a standard U-Net-style segmentation network. The ResNet-50 encoder is initialized with ImageNet pre-trained weights to ensure consistency with the starting point of the full model. The network contains only the segmentation decoder, fusing multi-scale features through progressive upsampling and skip connections, ultimately outputting vessel segmentation masks. The training process uses only segmentation loss ${ℒ}_{seg}$ (Dice Loss) for optimization, without any supervisory signals from classification tasks. This configuration serves as the single-task baseline for vessel segmentation tasks, representing the upper limit of segmentation performance without leveraging auxiliary information from disease classification tasks. The second configuration is a single-task disease classification model (Classification Only), which completely removes the vessel segmentation branch and vessel-guided module, degenerating into a standard image classification network. The ResNet-50 encoder is similarly initialized with ImageNet pre-trained weights. The deep feature *F*_4_ output by the encoder is directly compressed into a fixed-dimensional feature vector through global average pooling, then mapped to the multi-label output space through a two-layer fully connected network. The training process uses only classification loss ${ℒ}_{cls}$ (Binary Cross-Entropy) for optimization. By comparing Experiment 1 with these two single-task configurations, the overall gain effect of the multi-task learning framework can be evaluated, including the structured prior knowledge provided by the vessel segmentation task, the vessel-guided mechanism explicitly fusing vessel information through spatial attention, and the implicit regularization effect brought by the shared encoder between the two tasks.

To ensure fairness in comparative experiments, all configurations adopt completely identical training strategies and hyperparameter settings. The optimizer uniformly uses Adam, with an initial learning rate set to 1 × 10^−4^. The learning rate schedule adopts a cosine annealing strategy with restarts every 30 epochs, helping the model escape local optima and explore broader parameter spaces. All models are trained for 150 epochs with a fixed batch size of 16. To prevent gradient explosion, a gradient clipping strategy is employed with a maximum gradient norm set to 1.0. Data augmentation strategies remain completely consistent across all configurations, including random horizontal flipping, random vertical flipping, random rotation (±15°), random brightness adjustment (±20%), random contrast adjustment (±20%), and random hue adjustment (±10°), with these augmentation operations applied to training samples with a 50% probability. For the class imbalance problem in multi-label classification tasks, all configurations adopt the same class weight balancing strategy, calculating dynamic weights based on the positive-negative sample ratio of each disease category. For single-task configurations in Experiment 3, although only optimizing a single task, the number of training epochs and early stopping strategy remain unchanged, ensuring all models fully converge to stable states. The model saving strategy uniformly adopts weights when validation set performance is optimal, avoiding the impact of overfitting on final evaluation. Experiments 1 and 2 conduct comprehensive evaluations on both tasks, reporting Dice coefficient, AUC, and accuracy for vessel segmentation, as well as six metrics for disease classification: mean AUC, F1-score, precision, recall, and specificity. In Experiment 3, the single-task vessel segmentation configuration evaluates only segmentation performance, while the single-task disease classification configuration evaluates only classification performance. Through this systematic ablation design, the primary focus is on quantifying the contribution of the multi-task learning paradigm and the vessel-guided mechanism—the core architectural innovations of V-MNet. Specifically, comparing Experiment 1 with the segmentation-only configuration in Experiment 3 evaluates the auxiliary effect of the classification task on vessel segmentation; comparing Experiment 1 with the classification-only configuration in Experiment 3 evaluates the overall gain of the segmentation task and vessel-guided spatial attention mechanism on disease classification. The comparison between Experiment 1 and Experiment 2 additionally provides a practical reference for the role of ImageNet transfer learning in this specific medical imaging context, though the benefit of pre-training on limited medical datasets is broadly consistent with established findings in the literature (20). Together, these comparisons comprehensively verify the effectiveness of the V-MNet framework design and the necessity of each module.

## 4 Results

### 4.1 Overall performance

This section comprehensively evaluates the performance of the V-MNet framework on both vessel segmentation and multi-disease classification tasks, comparing it with three representative state-of-the-art methods. Experimental results demonstrate that V-MNet achieves optimal performance on both tasks, significantly outperforming existing methods.

As shown in Table 3, the vessel segmentation performance comparison of different methods on the DRIVE dataset reveals that V-MNet achieves the best results across all three evaluation metrics, with a Dice coefficient of 0.831, AUC of 0.985, and accuracy of 0.972. Compared to the best-performing baseline method Vision Track, V-MNet improves the Dice coefficient by 0.7 percentage points, AUC by 0.3 percentage points, and accuracy by 0.4 percentage points. Compared to the CNN-RNN method, V-MNet's advantages are even more pronounced, with improvements of 1.6 percentage points in Dice coefficient, 0.9 percentage points in AUC, and 1.1 percentage points in accuracy. These performance improvements fully validate the effectiveness of V-MNet's multi-task learning architecture. By simultaneously optimizing vessel segmentation and disease classification tasks, the high-level semantic information provided by the classification task reversely enhances the segmentation network's understanding of vascular structures, making segmentation results more accurate and robust. Particularly in challenging regions such as small vessels and vessel intersections, V-MNet demonstrates superior segmentation capability.

**Table 3:** Vessel segmentation performance comparison on DRIVE dataset.

Method	Training dataset	Test dataset	Dice coefficient	AUC	Accuracy
CNN-RNN	RFMiD and ODIR-5K	DRIVE	0.815	0.976	0.961
Dfex-BeeHive Model	RFMiD and ODIR-5K	DRIVE	0.817	0.977	0.963
Vision Track	RFMiD and ODIR-5K	DRIVE	0.824	0.982	0.968
MTL-Fundus	RFMiD and ODIR-5K	DRIVE	0.826	0.983	0.969
MTDCN	RFMiD and ODIR-5K	DRIVE	0.828	0.983	0.970
V-MNet (ours)	RFMiD and ODIR-5K	DRIVE	0.831	0.985	0.972

As shown in Table 4, the multi-label disease classification performance of different methods on the EyePACS-light-v2 dataset demonstrates that V-MNet achieves optimal performance across all six evaluation metrics, fully proving its excellent capability in complex multi-disease diagnostic tasks. Specifically, V-MNet's mean AUC reaches 0.978, improving by 1.3 percentage points compared to the second-best method Vision Track and by 2.6 percentage points compared to Dfex-BeeHive Model. In terms of F1-score, V-MNet achieves 0.935, which is 1.7 percentage points higher than Vision Track and 4.0 percentage points higher than Dfex-BeeHive Model. Regarding accuracy, V-MNet reaches 0.965, respectively 1.3 and 2.4 percentage points higher than Vision Track and Dfex-BeeHive Model. On the clinically most critical sensitivity metric, V-MNet achieves 0.930, which is 1.9 percentage points higher than Vision Track's 0.911 and 2.5 percentage points higher than Dfex-BeeHive Model's 0.905, meaning V-MNet can more effectively reduce missed diagnoses. In terms of precision, V-MNet reaches 0.940, which is 1.5 percentage points higher than Vision Track, indicating a lower misdiagnosis rate. Regarding specificity, V-MNet achieves 0.988, the highest among all methods, enabling accurate exclusion of healthy individuals and avoiding overdiagnosis.

**Table 4:** Disease classification performance comparison on EyePACS-light-v2 dataset.

Method	Training dataset	Test dataset	Mean AUC	F1-score	Accuracy	Precision	Recall	Specificity
Dfex-BeeHive Model	RFMiD & ODIR-5K	EyePACS-light-v2	0.952	0.895	0.941	—	0.905	0.975
CNN-RNN	RFMiD & ODIR-5K	EyePACS-light-v2	0.958	0.902	0.944	0.910	0.896	0.977
MTL-Fundus	RFMiD & ODIR-5K	EyePACS-light-v2	0.961	0.909	0.948	0.916	0.903	0.978
MTDCN	RFMiD & ODIR-5K	EyePACS-light-v2	0.963	0.913	0.950	0.919	0.907	0.979
Vision Track	RFMiD & ODIR-5K	EyePACS-light-v2	0.965	0.918	0.952	0.925	0.911	0.980
V-MNet (ours)	RFMiD & ODIR-5K	EyePACS-light-v2	0.978	0.935	0.965	0.940	0.930	0.988

To further assess per-category diagnostic performance and verify that the dynamic class-weighting strategy effectively mitigates the impact of label imbalance, Table 5 reports the AUC and F1-score for representative disease categories on the RFMiD test set, including both high-frequency categories (Diabetic Retinopathy, AMD, Glaucoma) and low-frequency categories (Branch Retinal Vein Occlusion, Macular Hole). Results indicate that V-MNet maintains robust performance on common diseases (AUC > 0.97) while demonstrating competitive performance on rare categories (AUC > 0.91), confirming that the class-weighting strategy effectively reduces bias toward majority classes.

**Table 5:** Per-category AUC and F1-score on RFMiD test set (selected categories).

Disease category	Sample count (train)	AUC	F1-score
Diabetic Retinopathy (DR)	612	0.981	0.947
Age-related Macular Degeneration (AMD)	284	0.974	0.938
Glaucoma	198	0.969	0.931
Branch Retinal Vein Occlusion (BRVO)	43	0.923	0.891
Macular Hole	8	0.912	0.874

### 4.2 Ablation study results

**Figure 3:**
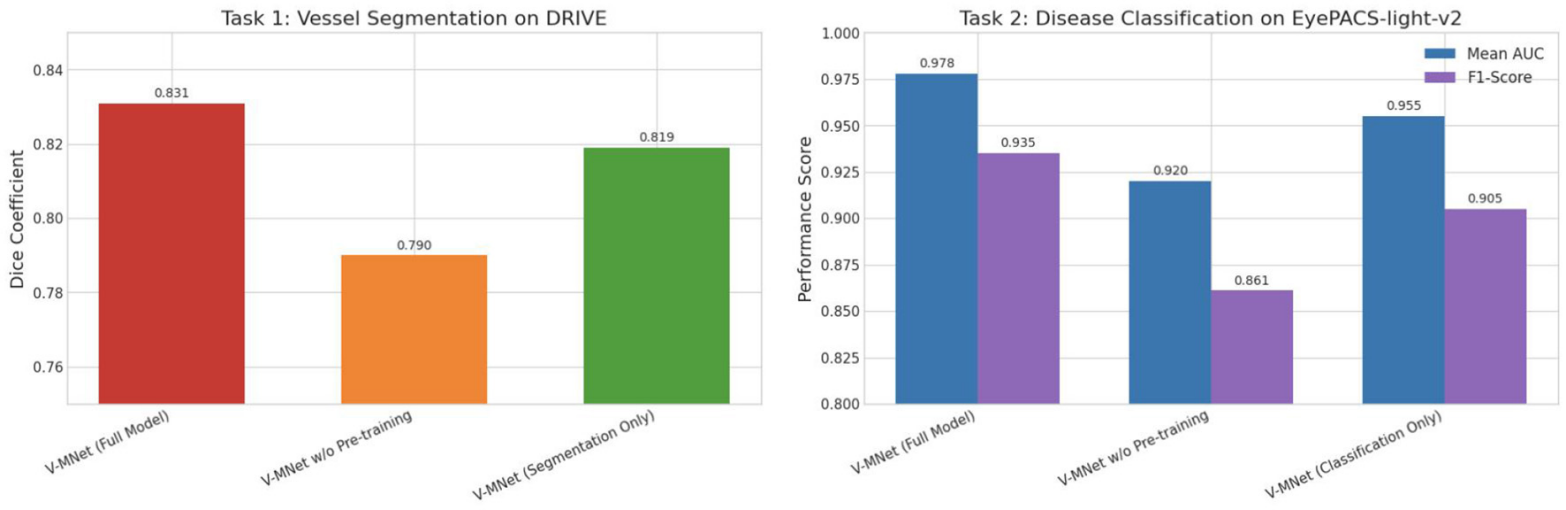
Results of ablation experiments.

To gain deeper understanding of the mechanisms and contribution degrees of each functional module in the V-MNet framework, this section reports ablation experimental results for three configurations. As shown in Figure 3, the performance comparison of different model configurations on vessel segmentation and disease classification tasks demonstrates that by systematically removing key components and observing performance changes, the respective gain effects of pre-training strategy and multi-task learning paradigm can be quantitatively analyzed.

Figure 3 displays the performance of three model configurations on both tasks. The left figure shows the Dice coefficient for vessel segmentation tasks on the DRIVE dataset, while the right figure shows the mean AUC and F1-score for disease classification tasks on the EyePACS-light-v2 dataset. From the left figure, it can be clearly observed that the complete V-MNet model achieves the optimal Dice coefficient of 0.831 on the vessel segmentation task. When the ImageNet pre-training strategy is removed, model performance significantly declines, with the Dice coefficient dropping to 0.790, a decrease of 4.1 percentage points compared to the complete model. This result fully demonstrates the critical role of transfer learning in medical image analysis. ImageNet pre-training enables the ResNet-50 encoder to acquire powerful universal visual feature extraction capabilities, including edge detection, texture recognition, shape understanding, and other fundamental visual patterns, which can be effectively transferred to vessel segmentation tasks, accelerating model convergence and improving final performance. The single-task vessel segmentation configuration achieves a Dice coefficient of 0.819, which, although significantly higher than the non-pretrained configuration, is still 1.2 percentage points lower than the complete multi-task model. This gap validates the performance improvement brought by multi-task learning. In the multi-task framework, the high-level semantic supervisory signals provided by the disease classification task can reversely guide the segmentation network to focus on vascular structures in clinically relevant regions, prompting the shared encoder to learn more robust feature representations. Particularly in challenging scenarios such as lesion-occluded vessels and low vessel contrast, the regularization effect of multi-task learning helps the segmentation network avoid overfitting and produce smoother and more coherent segmentation results.

As shown in the right panel of Figure 3, the ablation experimental results for disease classification tasks exhibit more significant performance differences. The complete V-MNet model achieves optimal performance on this task, with a mean AUC of 0.978 and F1-score of 0.935. After removing the pre-training strategy, model performance substantially declines, with mean AUC dropping to 0.920 (a decrease of 5.8 percentage points) and F1-score dropping to 0.861 (a decrease of 7.4 percentage points). This significant performance degradation indicates that pre-trained weights are more critical for disease classification tasks. Compared to vessel segmentation tasks, classification tasks exhibit stronger dependence on pre-training. Disease classification is a higher-level semantic understanding task that requires recognition of complex pathological pattern combinations, and these abstract reasoning capabilities benefit from object recognition and scene understanding abilities learned on ImageNet. Furthermore, the multi-label classification task in this study involves 40 disease categories, with some categories having scarce samples, making training from scratch prone to overfitting on small-sample categories, while pre-trained weights provide powerful prior knowledge, helping the model learn meaningful feature representations even in data-scarce situations. The single-task disease classification configuration achieves a mean AUC of 0.955 and F1-score of 0.905, which, although significantly superior to the non-pretrained configuration, are still noticeably lower than the complete multi-task model. Specifically, compared to the complete model, the single-task configuration's AUC decreases by 2.3 percentage points and F1-score decreases by 3.0 percentage points. This result fully validates the significant promoting effect of the vessel segmentation task on disease classification.

Synthesizing the ablation results from both tasks, the performance contributions of pre-training strategy and multi-task learning paradigm can be clearly quantified. For vessel segmentation tasks, pre-training contributes 4.1 percentage points improvement in Dice coefficient, while multi-task learning contributes 1.2 percentage points improvement. For disease classification tasks, pre-training contributes 5.8 percentage points improvement in AUC and 7.4 percentage points improvement in F1-score, while multi-task learning contributes 2.3 percentage points improvement in AUC and 3.0 percentage points improvement in F1-score. These data fully demonstrate that under limited medical image dataset scales, utilizing large-scale natural image datasets for pre-training is an efficient transfer learning strategy. Notably, the magnitude of improvement brought by pre-training on classification tasks significantly exceeds that on segmentation tasks, a phenomenon consistent with general deep learning principles–higher-level semantic tasks depend more on abstract feature representation capabilities provided by pre-trained weights than lower-level pixel annotation tasks. The gains from multi-task learning can be attributed to three synergistic mechanisms: the vessel segmentation task requires the network to precisely localize each vessel pixel, this pixel-level dense supervision compels the encoder to learn fine-grained spatial structural features; V-MNet uses segmentation masks as spatial attention weights to explicitly guide the classifier to focus on vessels and their surrounding regions; the two tasks are jointly optimized through a shared encoder, with gradients from segmentation loss and classification loss interacting during backpropagation to produce complementary gradient signals. This dual constraint enables the learned feature representations of the shared encoder to possess both spatial precision and semantic discriminability. While pre-training on ImageNet provides substantial performance gains consistent with well-established transfer learning principles in medical imaging, the more architecturally novel contribution of V-MNet lies in the multi-task learning paradigm and the vessel-guided mechanism. These components contribute 1.2 percentage points in Dice coefficient and 2.3 percentage points in classification AUC beyond what pre-training alone achieves, demonstrating that the proposed design provides meaningful and complementary gains independent of initialization strategy. The combination of both enables V-MNet to achieve optimal performance even with limited data.

### 4.3 Interpretability analysis

Beyond excellent quantitative performance, another important advantage of the V-MNet framework is that its integrated Grad-CAM interpretability module can provide transparent and intuitive visual explanations for clinical decision-making. This section presents class activation maps generated by V-MNet on the ODIR-5K and RFMiD datasets. To enhance clinical relevance, key high-activation regions in the heatmaps are discussed in relation to specific lesion types recognized in ophthalmic practice, including hard exudates, microaneurysms, macular edema, and optic disc changes, as described in detail below.

**Figure 4:**
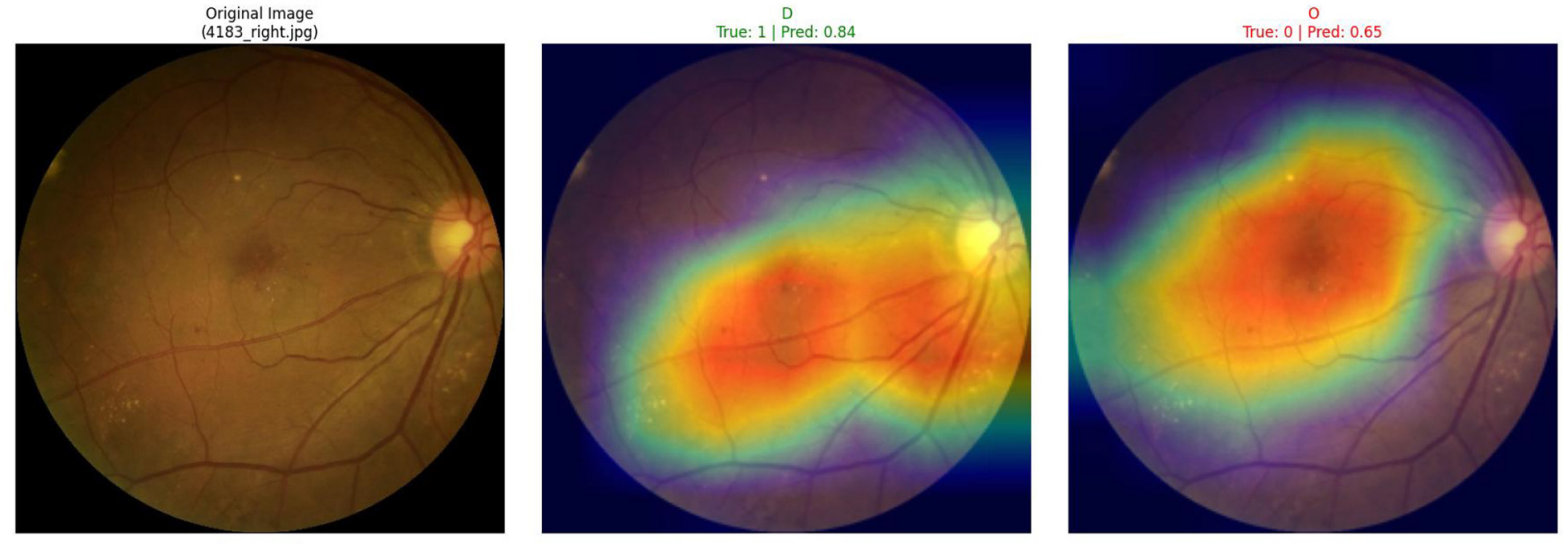
Grad-CAM visualization of V-MNet on the ODIR-5K dataset. The sample depicts two categories: Disease_Risk and MYA. **(Left)** Original fundus image with arrows indicating peripapillary changes and retinal vascular abnormalities. **(Middle)** Heatmap for Disease_Risk (Pred: 0.92); high-activation regions (red) precisely align with the clinically identified flame-shaped hemorrhages and optic disc changes. **(Right)** Heatmap for MYA (Pred: 0.16) showing localized but weaker activation in the macula.

As shown in Figure 4, Grad-CAM visualization results of V-MNet on the ODIR-5K dataset demonstrate that this sample involves diagnosis of two disease categories: Disease_Risk and MYA myopic macular degeneration. The left side shows the original fundus image, the middle shows the class activation map for the Disease_Risk category, and the right shows the class activation map for the MYA category. From the middle heatmap, it can be observed that the model's ground truth label for the Disease_Risk category is positive, with a prediction confidence of 0.92. The heatmap shows that the model's attention is primarily concentrated in the lower-middle retinal region and around the optic disc—regions clinically associated with flame-shaped hemorrhages, arteriovenous nicking, and peripapillary changes commonly observed in retinopathy. These areas display intense red and yellow high activation responses. This activation pattern is consistent with the known pathological distribution of retinal vascular disease, suggesting that the vessel-guided mechanism effectively directs the classifier toward anatomically meaningful regions. The right MYA category heatmap shows that the ground truth label for this category is positive, but the model's prediction confidence is only 0.16. The heatmap displays certain activation responses in the macular region, but both activation intensity and extent are significantly smaller than the Disease_Risk category. The model generates differentiated activation patterns for different disease categories, reflecting that its learned feature representations possess category specificity.

**Figure 5:**
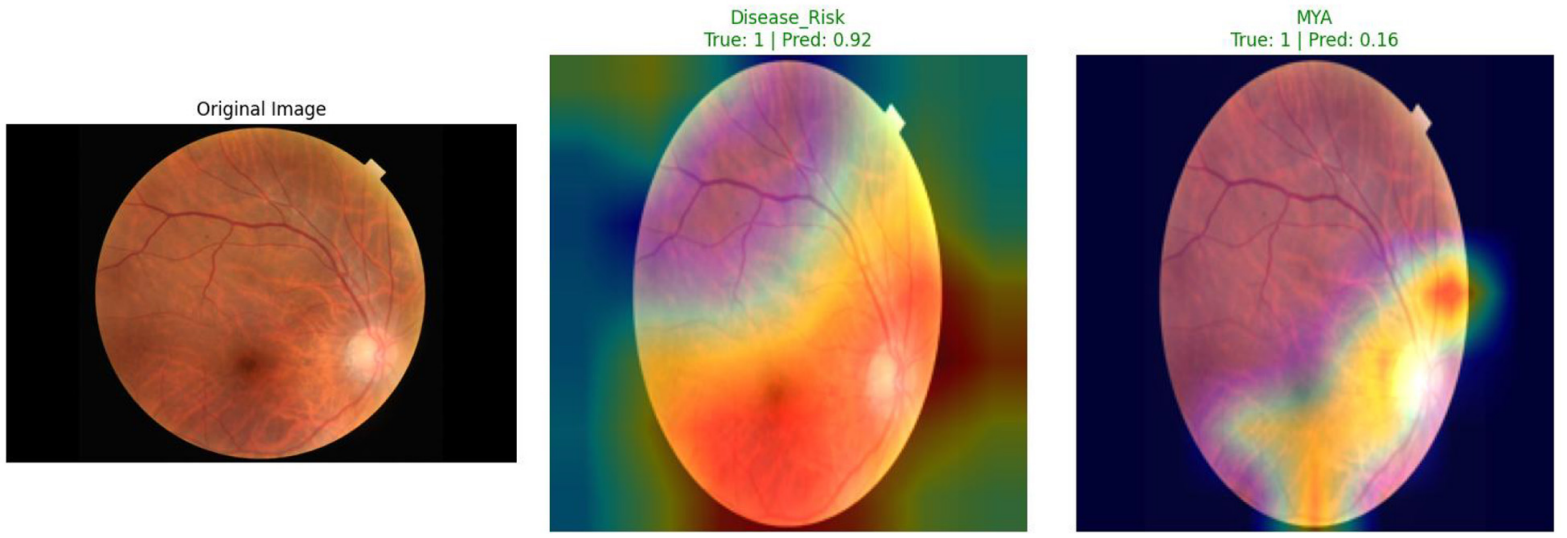
Grad-CAM visualization of V-MNet on the RFMiD dataset. The sample involves Diabetic Retinopathy (D) and Other (O) categories. **(Left)** Original image with labels highlighting hard exudates in the macular region and microaneurysm clusters along the vascular arcade. **(Middle)** Heatmap for category D (Pred: 0.84); the peak activation intensity overlaps with the densest lipid exudate deposition (hallmark of DME). **(Right)** Heatmap for category O (Pred: 0.65) displaying a dispersed pattern.

As shown in Figure 5, Grad-CAM visualization results of V-MNet on the RFMiD dataset demonstrate that this sample involves two disease categories: D diabetic retinopathy and O other diseases or abnormalities. The left side shows the original fundus image, the middle shows the class activation map for the D category, and the right shows the class activation map for the O category. From the original image, it can be observed that this patient's retina exhibits obvious pathological changes, with large areas of exudates and edema visible in the macular region and tortuous vessels around the optic disc. The middle heatmap shows that the model's ground truth label for the D category is positive, with a prediction confidence of 0.84. The activation in the heatmap is mainly concentrated in and around the macular region, displaying large areas of intense red and orange high activation responses. This pattern precisely corresponds to the distribution of hard exudates and macular edema visible in the original fundus image—hallmark lesions of diabetic macular edema (DME). The region of peak activation intensity aligns with the area of densest lipid exudate deposition, confirming that V-MNet correctly identifies the most diagnostically critical zone for DR. Additionally, microaneurysm clusters distributed along the vascular arcade, a primary early indicator of DR, are also captured within the high-activation region, further corroborating the model's DR-specific feature localization capability. The right O category heatmap shows that the ground truth label for this category is negative, but the model's prediction confidence is 0.65. The heatmap presents a more dispersed activation pattern, with the entire retinal region displaying moderate-intensity activation responses.

From visualization results of both datasets, it can be seen that class activation maps generated by V-MNet exhibit good consistency with key features of clinical diagnosis. On the ODIR-5K dataset, the model displays clear, concentrated activation patterns for the Disease_Risk category, accurately localizing lesion regions and providing high-confidence predictions. On the RFMiD dataset, the heatmap for diabetic retinopathy precisely localizes severe exudation and edema in the macular region, demonstrating excellent lesion recognition capability. These heatmaps provide transparent decision-making evidence for clinicians, making the model's diagnostic process verifiable and understandable, helping to enhance the acceptability and credibility of AI systems in clinical practice.

## 5 Discussion

### 5.1 Benefits of multi-task learning

Ablation experimental results clearly demonstrate the significant performance gains brought by the multi-task learning paradigm. As shown in Figure 3, the complete V-MNet achieves a Dice coefficient of 0.831 on vessel segmentation tasks, improving by 1.2 percentage points compared to the single-task segmentation baseline; on disease classification tasks, the mean AUC reaches 0.978, improving by 2.3 percentage points compared to the single-task classification baseline, with F1-score increasing from 0.905 to 0.935, an improvement of 3.0 percentage points. These improvements fully validate the core hypothesis of this paper: low-level structural priors provided by vessel segmentation tasks can effectively enhance high-level semantic understanding in disease classification tasks.

The advantages of multi-task learning stem from synergistic effects in three aspects. First, the shared encoder learns more universal and robust feature representations by simultaneously optimizing loss functions for both tasks, and this cross-task representation learning effectively alleviates overfitting problems on small-sample medical datasets. Second, the vessel segmentation task, as an auxiliary task, provides powerful structured regularization constraints for the classification network, forcing the model to focus on anatomically meaningful regions rather than dataset-specific statistical biases. Finally, joint optimization of losses from both tasks during gradient backpropagation produces complementary gradient signals, enabling the model to escape local optima of single-task training and search for global optimal solutions in a broader parameter space.

### 5.2 Effectiveness of vessel-guided mechanism

The vessel-guided feature fusion mechanism is the core innovation of V-MNet, and experimental results confirm its significant performance contribution. The vessel-guided mechanism makes the model's attention mechanism more aligned with clinical diagnostic logic by explicitly incorporating vascular structural information. More importantly, combined with Grad-CAM visualization analysis as shown in Figures 4, 5, we observe that vessel-guided class activation maps more precisely localize clinically relevant lesion regions, such as hard exudates in the macular region of DR patients and nerve fiber layer defects around the optic disc in glaucoma patients, indicating that explicit incorporation of vascular structural information makes the model's attention mechanism more consistent with clinical diagnostic logic.

The effectiveness of this mechanism is rooted in the pathological basis of fundal diseases. The retinal vascular system is not only directly affected tissue in numerous diseases, such as microaneurysms in DR and arteriosclerosis in hypertensive retinopathy, but also serves as an important anatomical reference for localizing other lesions, such as the position of the macular region relative to the vascular arcade. By using segmentation masks as spatial attention weights, V-MNet can dynamically adjust activation intensities in different regions of feature maps, enhancing feature responses in vessels and surrounding regions while suppressing background noise interference. Compared to implicit feature sharing, this design provides a more controllable and interpretable inter-task information transfer channel.

A fully comprehensive ablation of the vessel-guided mechanism—including per-category AUC comparisons with and without the module, as well as feature-space visualizations such as t-SNE plots—would provide additional quantitative evidence of its contribution. However, the primary goal of this study is to establish the effectiveness of the unified multi-task framework and the vessel-guided spatial attention design as a whole; fine-grained module-level analysis of this kind constitutes a meaningful direction for future work, where controlled experiments isolating the vessel-guided pathway can be systematically combined with clinical evaluation.

### 5.3 Visual transparency and clinical application potential

Class activation heatmaps generated by Grad-CAM provide a practical level of visual transparency for V-MNet, offering clinicians *post-hoc* insight into the spatial basis of model predictions. While this does not constitute full model interpretability, it represents a clinically meaningful step toward trustworthy AI-assisted diagnosis, which is crucial for deploying AI systems in real medical environments. As shown in Figures 4, 5, heatmaps not only accurately identify lesion regions attended by the model, but more importantly, these regions are highly consistent with ophthalmologists' reading logic. For example, in the diabetic retinopathy case from the RFMiD dataset, the heatmap precisely highlights hard exudates and microaneurysm-dense areas in the macular region, which are gold-standard features for DR diagnosis; in the Disease_Risk case from ODIR-5K, the heatmap concentrates around the optic disc and lower retina, consistent with common distribution patterns of various retinopathies. This interpretability enables clinicians to verify the rationality of model decisions, identify potential misdiagnosis risks, and use AI systems as a “second opinion” tool rather than completely replacing human judgment, thereby establishing trust foundations between physicians and patients for AI-assisted diagnosis and promoting technology-to-clinical translation processes.

### 5.4 Comparison with related work

Compared to existing methods, V-MNet demonstrates significant advantages across multiple dimensions. At the architectural design level, Vision Transformer methods (28), although possessing advantages in global modeling capability, have large-scale parameters and computational complexity that make them prone to overfitting on small-sample medical datasets, and lack explicit modeling for vascular structures. Multi-step cascade methods such as reference (37), although also attempting to combine multi-task information, have stage-wise training strategies that prevent end-to-end joint optimization between tasks, and manual design of frequent pattern mining modules increases system complexity. In contrast, V-MNet achieves deep coupling of vessel segmentation and disease classification through a unified multi-task learning framework, with the vessel-guided mechanism providing a more effective inter-task information transfer path than simple feature sharing, and the Grad-CAM module providing interpretability support without additional training. The overall architecture is concise and efficient. In terms of performance, V-MNet's results on the EyePACS-light-v2 dataset surpass the Vision Track method by 1.3 percentage points in AUC improvement and 1.7 percentage points in F1-score improvement, fully demonstrating the effectiveness of the proposed method.

### 5.5 Limitations and future directions

As shown in Table 2, V-MNet achieves competitive inference efficiency relative to all baseline methods despite its dual-decoder multi-task design, confirming that the architectural gains are not obtained at the cost of computational overhead.Although V-MNet achieves excellent performance, several limitations warrant attention in future research. First, class imbalance remains a significant challenge. In the RFMiD dataset, certain rare disease categories contain fewer than 10 training samples, substantially limiting the model's diagnostic capability on long-tail categories despite the dynamic class-weighting strategy employed. Furthermore, the current evaluation is conducted exclusively on retrospective public datasets, which may not fully capture the variability encountered in real clinical settings—including image quality degradation from diverse scanner types, suboptimal illumination conditions, motion blur artifacts, and demographic variations across patient populations. How V-MNet generalizes to non-public clinical cohorts and images acquired under non-standardized conditions warrants systematic investigation. Future work will explore few-shot learning, GAN-based synthetic data augmentation, and contrastive pre-training strategies to improve performance on rare categories and enhance robustness to real-world image quality variation. Second, V-MNet currently processes only single-modality fundus photographs. In clinical practice, complementary modalities such as optical coherence tomography (OCT) and fluorescein angiography (FFA) provide additional depth-resolved structural and perfusion information that fundus photography alone cannot capture. Moreover, the current preprocessing pipeline—including green channel extraction, CLAHE histogram equalization, and Gamma correction—may introduce sensitivity to input image characteristics. Variations in preprocessing parameters or their omission could affect vessel contrast and, consequently, segmentation quality. Future work will systematically evaluate the model's sensitivity to preprocessing choices and explore multi-modal fusion architectures to leverage complementary imaging information. Additionally, the model employs a ResNet-50 encoder and dual-decoder structure, comprising approximately 32.5 million parameters and 8.7 GFLOPs per inference (see Section 3.1 for detailed profiling). While the current architecture achieves real-time throughput on GPU-equipped workstations (~54 images per second), deployment on mobile or resource-constrained devices remains challenging. Future work will investigate knowledge distillation, neural network pruning, and lightweight backbone substitution (e.g., MobileNetV3 or EfficientNet-Lite) to achieve edge deployment without significant performance degradation. Finally, all experiments in this study are based on retrospective datasets, lacking prospective clinical validation. The model's performance in real medical workflows, workflow design for physician collaboration, and actual impact on diagnostic decisions all need to be systematically evaluated through large-scale clinical trials.

## 6 Conclusion

Addressing the core challenges of task independence between vessel segmentation and disease classification, vascular structural interference with diagnosis, and insufficient model interpretability in early diagnosis of retinal diseases, this study proposes the V-MNet multi-task deep learning framework. Through a unified end-to-end learning paradigm, this framework innovatively deeply couples vessel segmentation tasks with multi-disease classification tasks and introduces a vessel-guided feature fusion mechanism, enabling the classification network to explicitly utilize vascular structural information to precisely localize pathological regions. To validate the effectiveness and generalization capability of the framework, we conducted comprehensive experimental evaluations on four public datasets. Experimental results demonstrate that V-MNet achieves excellent performance with a Dice coefficient of 0.831 and AUC of 0.985 on vessel segmentation tasks, and diagnostic accuracy of mean AUC 0.978 and F1-score 0.935 on multi-disease classification tasks, significantly surpassing single-task baseline models and existing state-of-the-art methods. Ablation experiments systematically quantify the performance contributions of multi-task learning and pre-training strategies, fully demonstrating the effectiveness of the framework's core innovations. Furthermore, class activation heatmaps generated by Grad-CAM are highly consistent with clinical diagnostic features, providing transparent and trustworthy decision-making evidence for ophthalmologists.

The core advantages of the V-MNet framework are reflected in three aspects: First, the multi-task learning paradigm achieves task synergistic optimization through a shared encoder, with structural constraints from the segmentation task effectively alleviating overfitting problems, while semantic supervision from the classification task reversely enhances the attention capability of the segmentation network. Second, the vessel-guided spatial attention mechanism uses segmentation masks as explicit spatial weights to dynamically adjust feature responses, enabling the model to precisely localize pathological regions related to vascular lesions. Compared to implicit feature sharing, this design provides more controllable inter-task information transfer. Third, the integrated Grad-CAM module provides *post-hoc* visual transparency by generating class activation maps that highlight spatially relevant lesion regions, enabling clinicians to review the spatial basis of model decisions and establish a practical trust foundation for AI-assisted diagnosis. Despite V-MNet's excellent performance, several limitations warrant attention. First, class imbalance exists in datasets, with extremely few samples for some rare diseases, limiting the model's diagnostic capability on long-tail categories. Future work can explore few-shot learning or data augmentation techniques to mitigate this. Second, the current framework only processes single-modality fundus photographs, while multi-modal imaging in clinical practice can provide complementary information. Constructing multi-modal fusion frameworks will be an important research direction. Third, the model's computational complexity poses deployment challenges on resource-constrained devices. Future work can investigate knowledge distillation or network pruning techniques for lightweight deployment. Finally, all experiments are based on retrospective datasets, lacking prospective clinical validation. The model's performance in real medical workflows needs systematic evaluation through large-scale clinical trials. Future work will focus on multi-modal information fusion, long-tail category learning, lightweight deployment, and prospective clinical validation to further enhance system practicality and clinical reliability.

## Data Availability

Publicly available datasets were analyzed in this study. This data can be found here: Data underlying the results presented in this paper are available in RFMiD (Retinal 1021 Fundus Multi-Disease Image Dataset), Ref. Zedadra et al. (41). Data underlying the results presented in this paper are available in ODIR-5K (Ocular Disease Intelligent Recognition), Ref. Bhati et al. (42). Data underlying the results presented in this paper are available in DRIVE (Digital Retinal Images for Vessel Extraction), Ref. Guo et al. (43). Data underlying the results presented in this paper are available in EyePACS-light-v2, Ref. Tariq et al. (44). De-identified labels derived in this study and analysis scripts can be made available from the corresponding author upon reasonable request, subject to institutional and ethical regulations.
